# Early Electroencephalogram to Predict Severity of Injury in Infants With Abusive Traumatic Brain Injury

**DOI:** 10.1177/08830738251377152

**Published:** 2025-09-26

**Authors:** Natasha A. Varughese, Ali Hasan, Naomi Cohen, Deja Smith, Rebekah L. Clarke, Jillian Grapsy, Rana Said, Darryl Miles, Lakshmi Raman, Deepa Sirsi

**Affiliations:** 1 2Department of Neurology, Division of Pediatric Neurology, 14742 UT Southwestern Medical Center, Dallas, TX, USA; 2 Department of Pediatrics, Division of Critical Care, 12334 UT Southwestern Medical Center, Dallas, TX, USA; 3 1501 UT Southwestern Medical School, Dallas, TX, USA; 4 Department of Trauma Services, Children's Medical Center, Dallas, TX, USA; 5 Department of Radiology, Division of Pediatric Radiology, 12334 UT Southwestern Medical Center, Dallas, TX, USA; 6 Department of Pharmacology, Children's Medical Center, Dallas, TX, USA

**Keywords:** abusive head trauma, electroencephalographic (EEG) scoring, non-accidental trauma, pediatric traumatic brain injury, seizure burden

## Abstract

This study explored the hypothesis that specific electroencephalographic (EEG) findings may correlate with outcomes in children with abusive head trauma. A retrospective chart review was conducted on children <2 years of age who were hospitalized after suspected abusive head trauma between 2015 and 2021. Based on validated scoring systems, a primary analysis was performed to correlate background EEG scores against magnetic resonance imaging (MRI) scores and neurofunctional outcomes. A secondary analysis was performed to correlate seizure burden against MRI and neurofunctional outcomes. Significant positive correlations (Spearman rank) were discovered between background EEG scores and MRI scores (0.338, *P* = .011) as well as between background EEG scores and motor functional scores at 1-6 months (0.332, *P* = .017) and 7-12 months (0.386, *P* = .08). Seizure burden and sensory functional scores showed positive but statistically insignificant correlations. In conclusion, for children with abusive head trauma, background EEG is an early marker of prognosis whereas seizure burden requires additional study.

Abusive head trauma is the leading form of traumatic brain injury in young children.^1^ Per the Centers for Disease Control and Prevention,^2^ about a quarter of these victims die and the remainder often have lifelong disability from cognitive, motor, verbal, and sensory impairments, as well as epilepsy, spasticity, and/or behavioral problems.^3–6^ Although an infant's brain has some capacity for neural plasticity and the long-term outcomes can be difficult to predict,^4^ abusive head trauma is known to cause worse outcomes than accidental traumatic brain injury.^7,8^ Of course, from a public health perspective, the most important goal is prevention of the abusive head trauma altogether. However, once the trauma has tragically occurred, two items are still within the control of the medical team. First, it is critical to optimize neuroprotection (such as seizure management) during the acute phase of the recovery period. Then, given the variability in outcomes, it is also important to try to predict the degree of disability that these patients may experience later in life, because that will affect the types of therapy as well as the accommodations for which their caregivers must plan.

The cornerstone of practical management of developmental disabilities involves early intervention and proactive planning.^9^ The more specific information that can be provided to the patient's caregivers in the early recovery phase, the better the patient's and family's outcomes are expected to be.

Currently, the literature documents many markers of mortality in this patient population, but fewer markers exist for morbidity within the survivor group. Markers of mortality include an initial Glasgow Coma Scale of 3 to 5, presence of retinal hemorrhages, intraparenchymal bleed, cerebral edema, increased intracranial pressure, and an International Normalized Ratio  >1.3.^10–13^ Most of those metrics arise from the neurosurgery literature as aids to help determine which patients are appropriate for neurosurgical management. Fewer studies have attempted to elucidate markers of long-term disability prognosis in surviving patients. A small study from 1998 showed that younger patients (<6 months at time of trauma) tended to have poorer outcomes,^14^ and this was recently verified by a study that documented similarly poor outcomes in younger trauma patients at longer follow-ups, at 5 and 11 years of age.^6^ Two studies from the neurosurgery department of Memphis^15,16^ showed that both ischemic strokes and the requirement for neurosurgical interventions were very strong predictors of severe neurologic disability.

However, the long-term effects of abusive head trauma can range from severe motor disability to more subtle learning difficulties and behavioral problems. More granular predictors of prognosis for this specific patient population have not yet been widely reported.

Seizure burden, as a concept, is increasingly being studied as a marker of illness severity in infants^17^ and electroencephalographic (EEG) background is also frequently investigated for the same purpose.^18^ It is well established that seizures are related (both as a marker of injury and a cause of the disability) to worsened short- and long-term outcomes^19^ for all types of traumatic brain injury. In a study of 108 intensive care unit patients (both infants and older children with a variety of diagnoses) from the United Kingdom, suppressed interictal background was correlated with increased mortality, and both asymmetric background and prolonged seizures were associated with severe disability or death at 1 year postdiagnosis.^20^ In a cohort of pediatric traumatic brain injury patients (ages <18 years), certain findings (normal EEGs, reactivity, and intact sleep architecture) were shown to correlate with good outcomes at time of discharge.^21^ Although the incidence of seizures is variable in general pediatric traumatic brain injury populations, the specific features of abusive head trauma involve many risk factors for acute seizures and epilepsy. One-third of patients have ischemic stroke and/or hypoxic ischemic encephalopathy, most injuries involve subdural and other types of intracranial hemorrhage, and the inherent delayed presentation to care and repetitive injury often results in cortical atrophy and brain parenchymal volume loss.^14,22,23^

The actual incidence of seizures in this population reflects these risk factors: studies have documented seizure rates from 57% to 73% in this patient population.^24–26^ In a study comparing various types of pediatric traumatic brain injury, abusive head trauma was found to be a very significant predictor of seizures compared with other trauma types, with an odds ratio of 6.0.^27^ A small cohort of abusive head trauma patients in Ontario had worse outcomes in patients with higher maximum 1-hour seizure count,^28^ providing support for this concept.

The early identification and management of seizures during the initial hospitalization is critical for optimum management, and thus practice in many centers has shifted in recent years toward earlier initiation of EEG. There is great variability in the degree of encephalopathy and seizure burden on EEG monitoring, and prior studies have suggested that this information is a useful early marker for the patient's long-term prognosis.^20,28,29^ However, the simple presence or absence of subclinical seizures as an isolated metric has not been shown to correlate with 1-year developmental outcomes,^26^ suggesting that a more detailed analysis of the EEG data would be required for prognostic purposes. Also, EEG has not been sufficiently explored as a means to predict specific clinical outcomes in abusive head trauma.

Thus, to further elucidate and help expand on the role of EEG in abusive head trauma patients, a correlational analysis was attempted against neuroimaging and neurofunctional outcomes.

## Materials and Methods

A retrospective chart review of patients with abusive head trauma under 2 years of age was performed from 2015 to 2021 using the Trauma Registry at Children's Medical Center Dallas. The trauma registry includes all patients evaluated at the Children's Medical Center Dallas by the trauma surgery team and includes patients evaluated for concerns of physical abuse. The registry tracks consults (including consults to the child abuse pediatricians [CAP] at the hospital) and final diagnoses. The child abuse pediatricians team completes a full evaluation and makes an assessment that ranges from accidental injury or medical cause, nonspecific for abuse, or highly concerning for abusive injury. This assessment may change during the hospitalization based on a variety of new data. The child abuse pediatricians team final assessment (at the time of discharge) only is included in the trauma registry. For this study, children <2 years old that were evaluated by the child abuse pediatricians team for head injury and had a final diagnosis of highly concerning for abusive injury were included. RedCAP database was used for data entry. This study was approved by the institutional review boards of UT Southwestern Medical Center and Children's Medical Center. Given the retrospective nature of the study, informed consent of subjects was waived by these boards.

### Data Elements

The data elements derived from the chart included demographics (age and weight at time of trauma, ethnicity), prior medical history (especially presence or absence of prematurity and epilepsy), characteristics of emergency department course, trauma metrics, disposition from the emergency department, characteristics of floor and/or intensive care unit course if applicable (including ventilation time, need for vasopressors, sedatives, management of increased intracranial pressure, International Normalized Ratio value, neurosurgical interventions), findings of head imaging during hospitalization (computed tomography and/or magnetic resonance imaging), presence and duration of EEG during hospitalization, presence and number of seizure medications during hospitalization and at discharge, disposition from the hospital, developmental outcomes and outpatient medications documented in clinic notes during the first year post-discharge (scored during the time windows of 0-6 months and then 7-12 months post-trauma). The latter were obtained from any available descriptive clinic notes, most often those of the child abuse pediatricians team, the primary care physicians (often the foster care clinic), neurology, ophthalmology, and/or physical medicine and rehabilitation.

### Scoring System for Outcome Analysis

Background EEG activity was analyzed based on the EEG reports. It was categorized into scores using criteria previously published in critically ill neonates^30^ and children.^31^ For neonates, severe EEGs were defined as a low-voltage background (<20 μV), severe scarcity of expected graphoelements, or markedly prolonged interburst intervals. Lesser degrees of severity (mild or moderate) were determined by shorter prolongation of interburst intervals and fewer markers of dysmaturity. For children, severe EEGs consisted of burst suppression or very low voltage background (<20 μV); moderate EEGs demonstrated nonreactive slowing or dysfunction, periodic patterns, or lack of sleep architecture; and mild EEGs showed reactive slowing (focal or generalized), intact sleep architecture, or intermittent focal dysfunction.

MRI results were scored using a modified grading system for abusive head trauma^16^ validated by the pediatric neurosurgery group in Memphis, Tennessee. In this scale, grade I had only skull fracture, IIa had cerebral edema or intracranial hemorrhage *without* infarct or neurosurgical requirement, IIb had the same *with* infarct but still no neurosurgical intervention, IIIa had *no* infarct but did require neurosurgical intervention, and IIIb had both infarct and requirement of neurosurgical intervention. If the brain injury resulted in death prior to neurosurgical intervention, the patient was automatically placed in group IIIa or IIIb (depending on presence of infarct). Extraventricular device placement and devices for cerebrospinal fluid drainage were considered neurosurgical procedures for the purposes of this study, in accordance to the original published scoring system. In our study, the scores were derived from the radiology reports documented in the chart, and 10% (23 of the original 231 patients) were scored directly from the images themselves by a neuro-radiologist (RC) to verify accuracy.

Neurofunctional outcomes were scored based on clinical documentation at follow-up visits for these patients using the Functional Status Scale.^32^ The Functional Status Scale was chosen because it has been used in previously published studies to quantify outcomes at outpatient follow-up examinations,^33,34^ including in cohorts with abusive head trauma.^8^ In the clinic documentation available for this retrospective cohort, the motor and sensory components of the Functional Status Scale were most consistently documented, so these numbers were analyzed separately rather than the total Functional Status Scale scores. The motor component of the Functional Status Scale ranks from 1 to 4, with 1 being normal, 2 corresponding to 1 dysfunctional limb, 3 corresponding to 2 or more dysfunctional limbs, and 4 being poor head control. The sensory component of the scale also grades from 1 to 4, ranging from normal vision and hearing (score of 1) to complete blindness and deafness (score of 4). We chose to only analyze these components (motor and sensory scores) because they were found during analysis to be the only components that reliably tracked the injury sustained in this cohort. The data for these scores was derived from clinic notes available in the electronic medical record, especially those of the child abuse pediatricians team, physical medicine and rehabilitation, neurology, and ophthalmology. The Functional Status Scale scores were then grouped into categories of mild, moderate, and severe so that they could be used for Spearman ρ parametric analysis, as described by Pollack et al.^32^

In the first subset of primary analysis, background EEG scores were compared to MRI scores. In the second subset of primary analysis, background EEG scores were correlated with Functional Status Scale scores. For the primary outcomes, these 3 categories of data (scores from the MRIs, EEGs, and developmental outcomes) were compared using nonparametric analysis with Spearman ρ to calculate the correlation coefficient.

Seizure burden was calculated manually from the direct EEG data. It was measured in 2 ways: the maximum time spent in electrographic seizures per hour during the EEG recording as well as the total time of electrographic seizures during the entire recording.^35,36^ Interictal scores and seizure burden were calculated by a pediatric neurology resident (NV) from the original EEG tracings with the assistance of a pediatric epileptologist (DS).

A secondary analysis was then performed to correlate seizure burden against MRI and Functional Status Scale scores.

## Results

### Primary Findings

**Figure 1.**
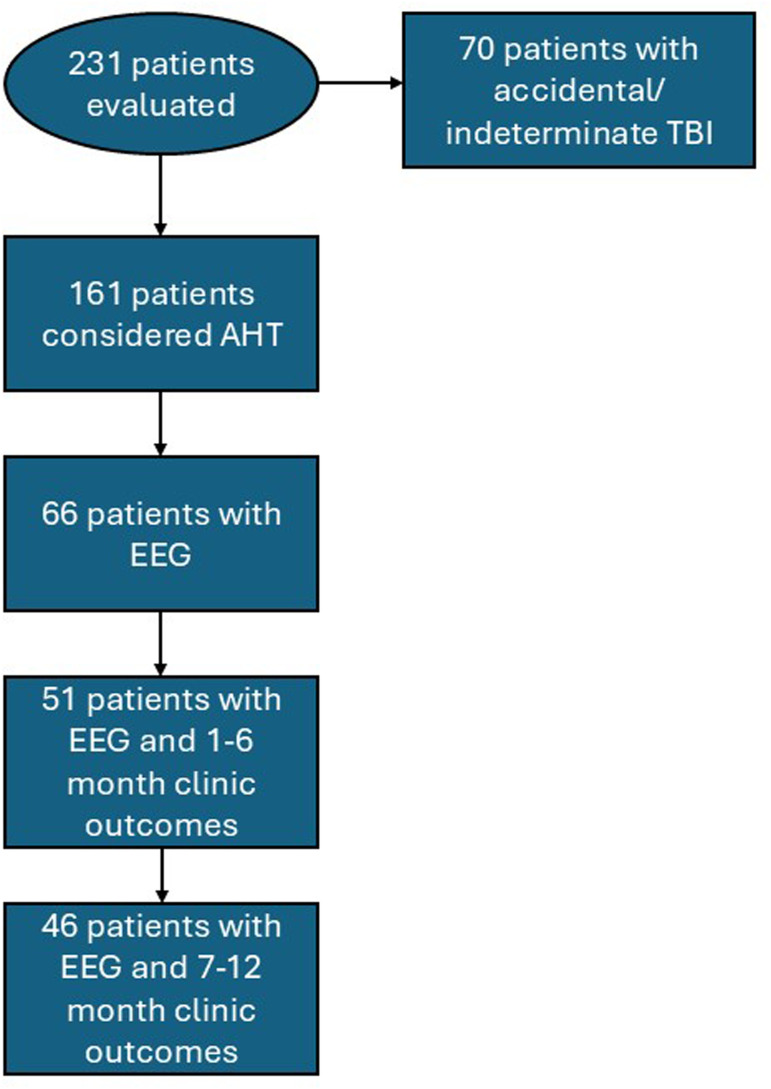
Flowchart of Patient Selection.

**Figure 2.**
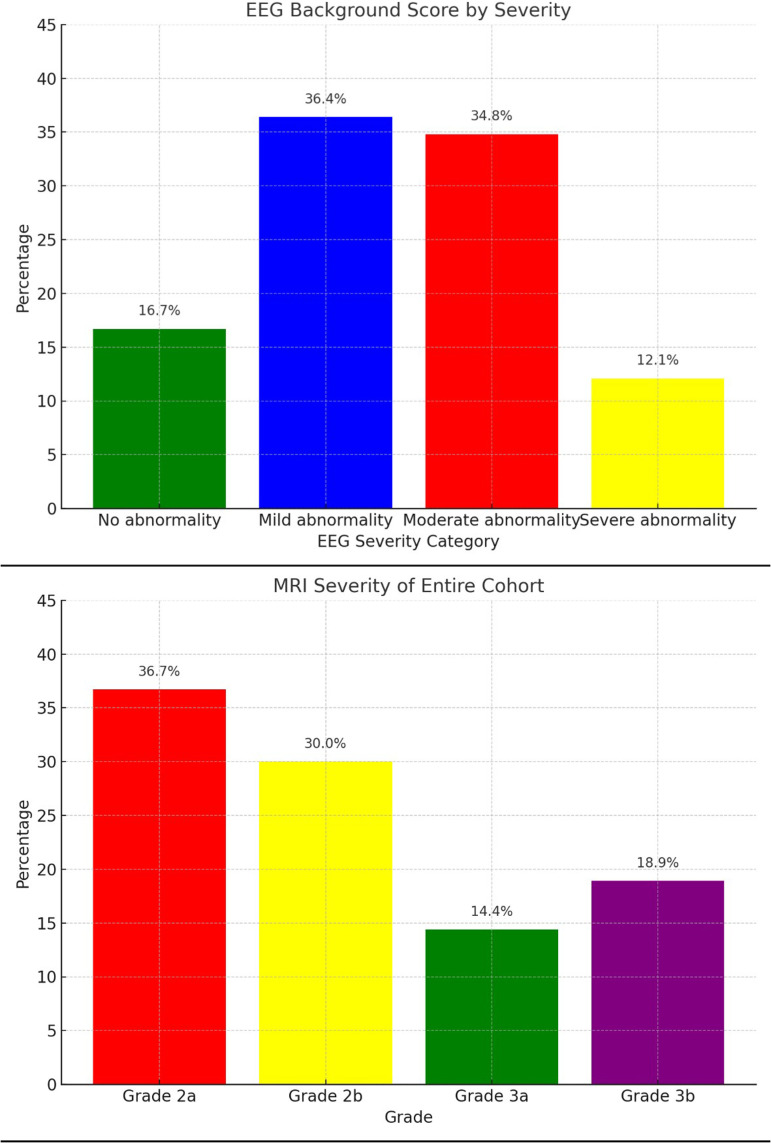
MRI Severity and EEG Background Score Categorized by Total Percentage of Patients. EEG, Electroencephalography; MRI, Magnetic Resonance Imaging.

Sixty-five EEGs were available to stratify by severity from the initial subset of selected patients (Figure 1). Interictal EEG scores were normal in 11 patients (17%), mildly abnormal in 23 (35%), moderately abnormal in 23 (35%), and severely abnormal in 8 patients (12%) (Figure 2).

Significant positive correlations were discovered between severity of background interictal EEG scores and MRI scores (0.338, *P* = .011) as well as severity of background interictal EEG scores and motor outcomes by Functional Status Scale at 1-6 months (0.332, *P* = .017) and 7-12 months (0.386, *P* = .008) (Table 1).

**Table 1.** Correlations Between EEG Background Score, Total Seizure Burden, Maximum Seizure Burden, and Both Imaging and Developmental Outcomes by Spearman ρ (Correlation Coefficient, Significance, and n).^a^

	Brain MRI score	FSS motor (0-6 mo)	FSS sensory (0-6 mo)	FSS motor (7-12 mo)	FSS sensory (7-12 mo)
EEG background score	**0.338, 0.011 (n** **=** **56)**	**0.332, 0.017 (n** **=** **51)**	0.218, 0.125 (n = 51)	**0.386, 0.008 (n** **=** **46)**	0.235, 0.116 (n = 46)
Total seizure burden (continuous variable)	0.213, 0.366	0.370, 0.108	0.432, 0.057		
Maximum seizure burden per hour (<20% vs >20%)	−0.135, 0.571	0.228, 0.335	0.370, 0.109		
Electrographic status epilepticus	−0.234, 0.320	0.189, 0.424	0.307, 0.187		

Abbreviation: FSS, Functional Status Scale.

a Bolded results are statistically significant.

Seizure burden and sensory functional scores showed positive but insignificant correlations, possibly because of the small number of patients available for analysis (Table 1).

Of the patients who obtained EEG while inpatient (n = 66), 27 had EEG results stored at our institution available for direct review by the authors. Based on this review, about one-third (36%) of the patients had electrographic status epilepticus (>50% seizure burden) at some point during the EEG performance. A much larger fraction (75%) had >20% seizure burden at some point during the recording. On average, the highest seizure burden for each patient (as defined by duration of seizures in a given hour) was 26 ± 16 minutes. The average total seizure burden (total seizure time for the entire EEG recording) was 116 ± 98 minutes. Total seizure burden did not have significant linear correlations with either the total length of hospitalization or the length of the intensive care unit stay.

Subclinical seizures were found in 48% of the patients with EEG studies (Table 2). However, none of the patients in our cohort had subclinical seizures without some clinical seizures.

**Table 2.** EEG Metrics (n = 27^a^).

EEG metric	Value (% or mean ± SD)
Electrographic status epilepticus (>50% seizures in 1 h), %	36
Maximum seizures per hour >20% (>12 min in 1 h) , %	75
Average maximum seizures per hour, min, mean ± SD	26 +/-16
Average total seizure burden, min, mean ± SD	116 +/-98
Background EEG score, %	
Normal	17
Mild	35
Moderate	35
Severe	12

Abbreviation: EEG, electroencephalography; SD, standard deviation.

a Patients with seizures who had EEG available for review by the authors.

Seizure medications were prescribed 1 year after discharge in about twice as many patients in the EEG cohort (55%) than in the general cohort (23%).

We did note the subset of patients who had EEG testing (66 of 161 patients) had essentially the same demographic characteristics as the larger cohort in terms of their history prior to injury, including gender, age at time of injury, and medical history. However, this subset of patients with EEG differed from the larger cohort mainly in the acuity of their illness during the hospitalization. This was reflected in the average length of their hospitalization (11.5 days) and intensive care unit stay (6 days), likelihood of requiring higher level of intensive care such as intubation (55%), or intracranial pressure management (27%) (Table 3).

**Table 3.** Comparative Analysis of Entire Cohort Versus Cohort With EEG Performed.

Demographic variable	Entire cohort	Cohort with EEG
Total n	161	66
Age, mo, mean ± SD	4.3 ± 2.8	3.5 ± 2.5
Sex (% female)	42	46
Weight, kg, mean ± SD	6.5 ± 2.1	6.1 ± 2.1
Prematurity (<37 wk), %	19	21
Clinical variable	Entire cohort (n = 161)	Cohort with EEG (n = 66)
GCS score on arrival 3-8, %	18	33
GCS score on arrival 9-12, %	5	11
GCS score on arrival 13-25, %	77	56
Total hospitalization duration, d, mean ± SD	6.8 ± 8	11.5 ± 10
ICU duration, d, mean ± SD	3.0 ± 4.8	6.2 ± 5.8
Required intubation, %	29	55
Required vasoactive medications, %	14	26
Required ICP management, %	11	27
Required neurosurgical intervention, %	25	39
Retinal hemorrhages present, %	38	64
Mortality, %	8	9
Required anti-seizure medications 1 y postdischarge, %	23	52
Required spasticity medications 1 y postdischarge, %	2	6
FSS scores, %	Entire cohort	EEG performed cohort
1-6 mo	n = 115	n = 66
Normal	59	45
Mild	8	12
Moderate	17	18
Severe	16	25
7-12 mo	n = 81	n = 45
Normal	51	33
Mild	12	15
Moderate	25	29
Severe	12	22
MRI findings, %	Entire cohort	Cohort with EEG
Brain MRI obtained	59	85
Subdural hemorrhage	95	83
Subarachnoid hemorrhage	23	24
Parenchymal hemorrhage	15	18
Acute ischemic injury	47	56
Diffuse axonal injury	11	17
MRI scores, %		
I	0	0
IIa	37	17
IIb	30	39
IIIa	14	14
IIIb	19	28

Abbreviations: EEG, electroencephalography; FSS, Functional Status Scale; GCS, Glasgow Coma Scale; ICP, intracranial pressure; ICU, intensive care unit; MRI, magnetic resonance imaging; SD, standard deviation.

### Secondary Findings

**Figure 3.**
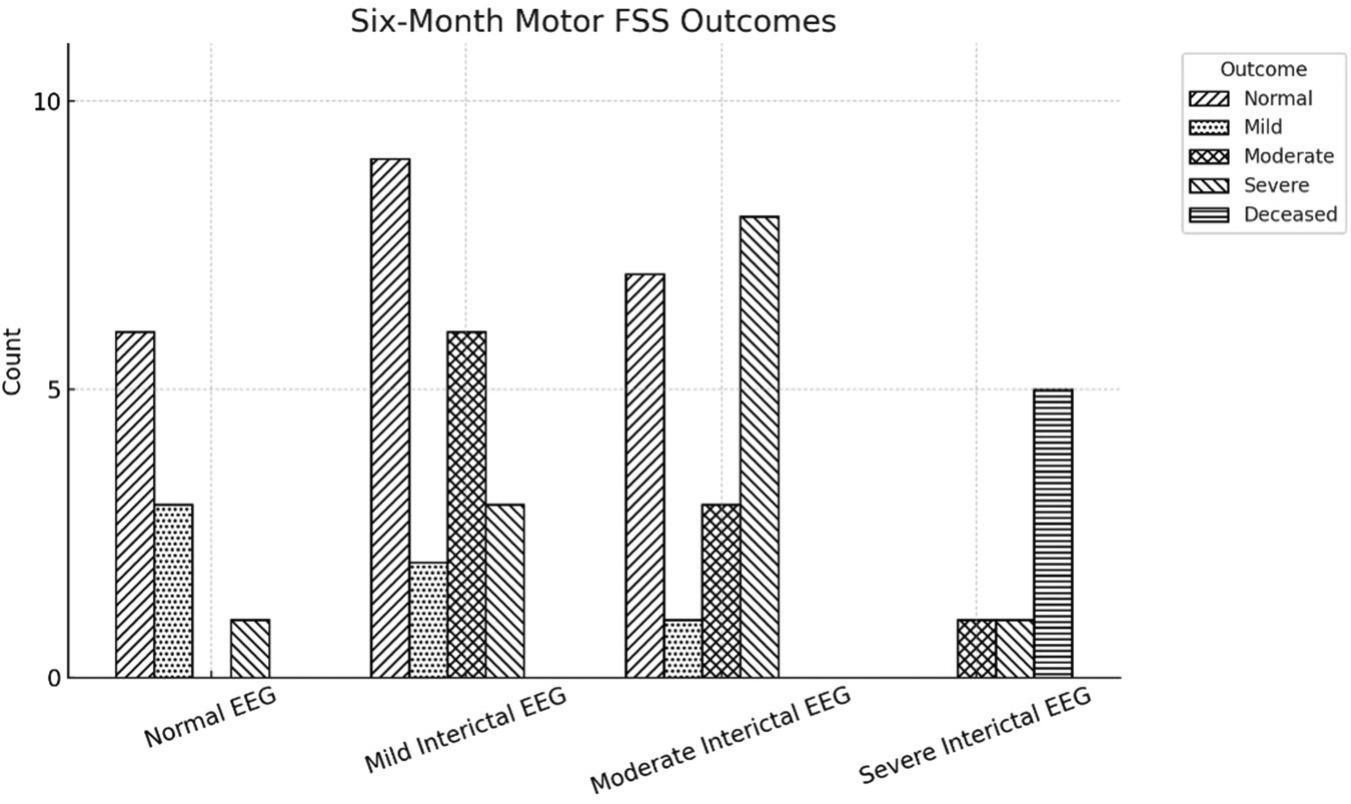
Correlations Between EEG Background and Motor Functional Status Scale Scores at the 0-6-Month Time Frame. EEG, Electroencephalography.

Brain MRI was obtained during initial hospitalization in 59% of the total cohort, and the most common findings were subdural hemorrhage (95%) and acute ischemia (47%). In the cohort with EEG, 86% of patients obtained brain MRI during the hospitalization, and the distribution of common findings was similar between the patients who obtained EEG and those who did not. Specifically, the rates of subdural hemorrhage, subarachnoid hemorrhage, parenchymal hemorrhage, and acute ischemia were similar between the groups with and without EEG. Timing of the initial brain MRI ranged from day 1 to 18, with an average 3.5 ± 2.8 days. In the cohort of patients who obtained EEG, there were zero patients with an MRI score of I, 11 (17%) with a score of IIa, 26 (39%) with a score of IIb, 9 (14%) with a score of IIIa, and 18 (28%) with a score of IIIb (Figure 3).

## Discussion

Our findings validate the hypothesis that early interictal EEG may be a reliable marker of neurologic injury and prognosis in patients with abusive head trauma. We found strong correlations between the EEG background and the MRI scores as well as motor neurologic outcomes. Although this study did not find a statistically significant correlation between seizures and outcomes, perhaps because of sample size, it did show a trend and it has been correlated with outcomes in prior studies.^37,38^ Granild-Jensen et al^28^ showed that a normal EEG background was correlated to a good outcome, but our study also showed with statistical significance that degrees of severity in interictal EEG are also predictive of outcome severity. This result, although intuitive, is an important datum for the purposes of counseling the families and planning patient care.

An important aspect of our study was an attempt to validate seizure burden (by 3 different metrics) as an independent marker of severity in this patient population using a relatively detailed grading scale for developmental outcomes. The seizure burden of our patient population did not show any correlations with imaging or functional outcomes, but this could also be due to the small sample size in that cohort. Greiner et al^26^ had attempted to correlate developmental outcomes in their cohort with the presence or absence of nonconvulsive status epilepticus, but that study also appeared to be limited in statistical power by the retrospective nature of the study and unreliable follow-up data. Granild-Jensen et al^28^ had demonstrated significant correlation between 1-hour seizure burden and mortality. Notably, that cohort was more severely injured than ours (23% mortality vs our 8%). Several older studies^26,29^ have established the prognostic value of very broad features of the EEG, such as asymmetry and burst suppression to predict mortality, “prolonged seizures” leading to a neurologically impaired outcome, and a combination of these features associated with even poorer outcomes, but these EEG features are not quantitative, and the findings were validated in a very diverse patient population with a wide variety of unrelated brain pathologies. In Barlow et al,^29^ the simple presence or absence of seizures predicted disability in nonaccidental trauma patients at follow-up, but again, the quantitative seizure burden or the degree of developmental disability was not described. Given the rising recognition of the importance of seizure burden^19,35^ as well as the unique nature of this individual patient population, careful elucidation of these metrics will be important to establish.

The demographics and medical history of the patients with EEG paralleled those characteristics of the rest of the cohort as well as the existing literature in the field,^16,23^ helping to validate the generalizability of the results. The spectrum of severity in imaging and EEG are comparable to the Cincinnati study,^26^ further validating our findings.

Over the time period listed for the retrospective data review our institution did not have a uniform policy on which patients were placed on EEG. On clinical review, EEGs were generally performed on patients after abusive head trauma who either had clinical seizure activity or on those patients whose clinical examination was compromised because of the need for sedation/invasive mechanical ventilation. This may reflect indirectly in our results where a higher proportion of patients placed on EEG needed invasive mechanical ventilation and higher tiers of intracranial pressure therapy. We further note that although subclinical seizures were found in a substantial proportion of our patients (48%), none of the patients in our cohort had subclinical seizures without some clinical seizures, perhaps reflecting the fact that EEG was generally only ordered in response to clinical seizures. At this institution, the trends documented in this study influenced the department to shift clinical practice toward automatically obtaining EEG in all patients admitted with concern for abusive head trauma. We hope that publishing the high incidence of subclinical seizures in this population will assist other centers, especially lower-resource institutions, in triaging the urgency of acute EEG for these patients.

The EEG ordering pattern did *not* correlate with the types of bleeding on brain MRI, suggesting that the presence of these MRI findings is not necessarily a reliable metric for predicting likelihood of seizures in this population. The one finding on MRI roughly associated with clinical seizures was the presence of acute ischemia, suggesting that EEG should be prioritized in abusive head trauma patients with this finding.

One limitation of the study is that the subset of patients with EEG tended to be more medically ill during their initial hospitalization, raising the concern that the worsened developmental outcomes in our results are simply a reflection of the fact that EEGs were only obtained in the more medically complex sample. In the future, a prospective study that performs EEG on all patients regardless of acuity may help better elucidate implications of EEG on prognosis. Another limitation is the fact that the study only followed the patients for 1 year posttrauma, which probably does not capture the full extent of the patients’ future disability. “Mild” presentations have the potential to exhibit learning disabilities and behavioral difficulties as they grow older,^6^ and the population of abusive head trauma patients are known to worsen over time more significantly than general pediatric traumatic brain injury patients,^8^ so longer-term follow-up of this patient population will be necessary for optimal assessment of prognosis. A prospective study with real-time analysis of seizure burden that allows for guided intervention might allow for an analysis on its impact on short- and long-term outcomes. Finally, calculation of seizure burden was done retrospectively, which is time consuming and subject to possible bias. In future prospective studies, this calculation needs to be done at the time of EEG to guide treatment decisions and prognosis. Availability of quantitative EEG technology, automated seizure detection software, and artificial intelligence technology would assist with this.

## Conclusion

In infants with abusive head trauma, several components of EEG may be useful as an early marker of prognosis. Higher background EEG scores correlated with worse MRI scores and neurologic outcomes during their first year posttrauma. Although this study did not find a statistically significant correlation between seizures and outcomes, perhaps because of sample size, it did show a trend and it has been correlated with outcomes in prior studies. This article provides preliminary evidence that EEG can be reliably used as a tool in counseling families on a patient's long-term prognosis in this specific population, but a larger prospective study would be required to validate these results.

## Supplemental Material

sj-docx-1-jcn-10.1177_08830738251377152 - Supplemental material for Early Electroencephalogram to Predict Severity of Injury in Infants With Abusive Traumatic Brain Injury Supplemental material, sj-docx-1-jcn-10.1177_08830738251377152 for Early Electroencephalogram to Predict Severity of Injury in Infants With Abusive Traumatic Brain Injury by Natasha A. Varughese, Ali Hasan, Naomi Cohen, Deja Smith, Rebekah L. Clarke, Jillian Grapsy, Rana Said, Darryl Miles, Lakshmi Raman and Deepa Sirsi in Journal of Child Neurology
