# Supplementary material for: Early Electroencephalogram to Predict Severity of Injury in Infants With Abusive Traumatic Brain Injury
Source: J Child Neurol. 2025 Sep 26;41(5):704–14. doi: 10.1177/08830738251377152 (PMC13069134; doi:10.1177/08830738251377152)
Supplement: sj-docx-1-jcn-10.1177_08830738251377152 - Supplemental material for Early Electroencephalogram to Predict Severity of Injury in Infants With Abusive Traumatic Brain Injury [file sj-docx-1-jcn-10.1177_08830738251377152.docx]

**Table of Contents:**

Page 2. S1. Table showing MRI Scoring System

Page 3-4. S2. Table showing EEG Interictal Background Scoring System (from Tharp and Laboyrie, 1983 as well as Fox et al, 2020)

**S1. Imaging Scoring System (reproduced with permission from Boop et al., 2016)**

| **Grade** | **Description** |
| --- | --- |
| I | Skull fracture alone, with or without soft tissue injury |
| IIa | Intracranial hemorrhage or cerebral edema NOT requiring neurosurgical intervention. NO infarct seen. |
| IIb | Intracranial hemorrhage or cerebral edema NOT requiring neurosurgical intervention. Infarct IS seen. |
| IIIa | Intracranial hemorrhage or cerebral edema that DOES require neurosurgical intervention. NO infarct seen. |
| IIIb | Intracranial hemorrhage or cerebral edema that DOES require neurosurgical intervention. Infarct IS seen. |

Boop, S., Axente, M., Weatherford, B., & Klimo, P., Jr (2016). Abusive head trauma: an epidemiological and cost analysis. *J Neurosurg Pediatr*, *18*(5), 542–549.

**S2. EEG Interictal Background Scoring System. Reproduced with permission from Fox et al, 2020. As well as Tharp and Laboyrie, 1983.**

**Neonatal EEG Severity Scoring.**

| **Mild** | **Moderate** | **Severe** |
| --- | --- | --- |
| Intermittent interhemispheric asymmetry | Excessive asynchrony and/or discontinuity | Isoelectric |
| Mild focal abnormalities, mild disturbance in background | Moderate persistent asymmetry | Marked increased interburst intervals |
| Mild excessive interhemispheric asymmetry | Focal sharp waves, spikes, or delta waves in addition to other abnormlities | Paroxysmal background with or without excessive interhemispheric asynchrony |
| Mild excessive discontinuity | Dysmaturity | Low voltage (<20 uV), diffusely slow background |
| Increase in frontal sharp waves | Occasional positive Rolandic sharp waves with abnormal background | Abundant positive Rolandic sharp waves |
|  | Excessive diffuse background delta activity with excessive discontinuity |  |

**Childhood EEG Severity Scoring**

| **Mild** | **Moderate** | **Severe** |
| --- | --- | --- |
| Intermittent generalized slowing | Continuous unreactive generalized slowing | Electrocerebral inactivity |
| Continuous reactive generalized slowing | No stage 2 sleep features seen | Burst suppression |
| Stage 2 sleep features seen | Periodic pattens or rhythmic discharges (LPDs, BPDs, triphasics, continuous spikes, etc.) | Very low voltage (< 20 uV) |
| Mild/intermittent focal slowing | Continuous focal asymmetry |  |
| Mild/intermittent focal asymmetry |  |  |
| Rare/Occasional/Infrequent interictal epileptiform discharges |  |  |

Fox, J., Jenks, C. L., Farhat, A., Li, X., Liu, Y., James, E., Karasick, S., Morriss, M. C., Sirsi, D., & Raman, L. (2020). EEG is A Predictor of Neuroimaging Abnormalities in Pediatric Extracorporeal Membrane Oxygenation. *J Clin Med*, *9*(8), 2512.

Tharp, B. R., & Laboyrie, P. M. (1983). The incidence of EEG abnormalities and outcome of infants paralyzed with neuromuscular blocking agents. *Crit Car Med*, *11*(12), 926–929.
